# Influence of the Chemical Structure on Odor Qualities and Odor Thresholds of Halogenated Guaiacol-Derived Odorants

**DOI:** 10.3389/fchem.2017.00120

**Published:** 2017-12-18

**Authors:** Florian Juhlke, Katja Lorber, Maria Wagenstaller, Andrea Buettner

**Affiliations:** ^1^Professorship of Aroma Research, Department of Chemistry and Pharmacy, Emil Fischer Center, Friedrich-Alexander-Universität Erlangen-Nürnberg, Erlangen, Germany; ^2^Department of Sensory Analytics, Fraunhofer Institute for Process Engineering and Packaging IVV, Freising, Germany

**Keywords:** gas chromatography-olfactometry, odorant, retention index, 5-chloroguaiacol, 5-bromoguaiacol, non-intentionally added substances (NIAS)

## Abstract

Chlorinated guaiacol derivatives are found in waste water of pulp mills using chlorine in the bleaching process of wood pulp. They can also be detected in fish tissue, possibly causing off-odors. To date, there is no systematic investigation on the odor properties of halogenated guaiacol derivatives. To close this gap, odor thresholds in air and odor qualities of 14 compounds were determined by gas chromatography-olfactometry. Overall, the investigated compounds elicited smells that are characteristic for guaiacol, namely *smoky, sweet, vanilla-like*, but also *medicinal* and *plaster-like*. Their odor thresholds in air were, however, very low, ranging from 0.00072 to 23 ng/L_air_. The lowest thresholds were found for 5-chloro- and 5-bromoguaiacol, followed by 4,5-dichloro- and 6-chloroguaiacol. Moreover, some inter-individual differences in odor threshold values could be observed, with the highest variations having been recorded for the individual values of 5-iodo- and 4-bromoguaiacol.

## Introduction

During processing of all sorts of raw materials in every industrial sector several undesired by-products can be formed. Those unwanted side products are mainly caused by physical or chemical reactions, and may be comprised within the term non-intentionally added substances (NIAS) (Geueke, 2015). Besides the formation of odorants in food or during food processing, odor active compounds can be built in non-food production as well. While in food industry the development of aroma compounds is a desired process, or at least controlled when it comes to the formation of off-odors, development of smell is commonly an unintended process in other industries. Smells in materials and products that are expected to be odorless may result in complaints or rejection by consumers. Further, those unwanted odor-active substances may be of physiological concern, be it for the consumer or the environment, as is the case for chlorinated phenols. These compounds are considered toxic in the respective relevant concentrations, and are critical with regards to biodegradation; Accordingly, they are listed amongst a group of priority toxic pollutants by the US EPA in the Clean Water Act and by the European Decision 2455/2001/EC. Moreover, the maximum allowed concentration of phenol, the base frame of halogenated guaiacols, in tap water is set at 0.05 μg/L by the European Union legislation (Hamad et al., 2010; Malhotra et al., 2013). Chlorophenolic compounds can easily penetrate skin and epithelium, which can lead to damage and necrosis, and employees working in phenoxyherbicides and chlorophenol producing industries more often come down with heart disease, asthma and lung cancer (Michałowicz, 2010).

As discussed in our previous publication on alkylated, alkenylated, and methoxylated guaiacol derivatives (Schranz et al., 2017), 2-methoxyphenol (guaiacol) is a decomposition product of wood, or lignin in particular, being formed in the course of thermal treatment (Kibet et al., 2012). Chlorinated guaiacol derivatives are potentially formed during bleaching of pulp and wood pulp, as a result of degradation and chlorination of lignin and its derivative guaiacol. In agreement with this, different chlorinated guaiacols, like 4-chloro-, 5-chloro-, 6-chloro-, or 4,5-dichloroguaiacol, could be identified in bleaching liquors, bleaching filtrates, and pulp mill wastewater or river sediments, surrounding pulp and paper mills, processing pine wood, eucalyptus, rice straw, wheat straw, or bamboo (Sequeira and Taylor, 1991; Owens et al., 1994; Judd et al., 1996; Sharma et al., 1997; Sinkkonen et al., 1997; Sharma and Kumar, 1999; Furtado João et al., 2000). Further, 4-chloro- and 4,5-dichloroguaiacol were detected in fish tissue of animals living downstream of bleached-kraft pulp mills (Owens et al., 1994; Araki et al., 2001). In general, the accumulation of chlorinated phenols in aquatic animals might endanger their health and that of their potential consumers (Pellinen et al., 1993; Michałowicz, 2010). Furthermore, 4,5-dichloroguaiacol has been identified in insects living in the vicinity of pulp mills (Owens et al., 1994).

Apart from that, several studies focused on strategies for industrial removal and biodegradability of chlorinated phenols, or chlorinated guaiacols in particular, via different approaches. Recently it was shown that 19.7% 4-chloroguaiacol and 13.5% 4,5-dichloroguaiacol can be removed from paper mill wastewater using poly-aluminum chloride as coagulant (Choudhary et al., 2015). Furthermore, 4-chloroguaiacol can be efficiently removed from aqueous solutions by high surface area of oil palm shell-activated carbon, activated with sodium hydroxide (Hamad et al., 2010). Gonzales et al. further demonstrated that 4-chloro-, 5-chloro-, 6-chloro-, and 4,5-dichloroguaiacol can be metabolized by *Acinetobacter junii* strain (González et al., 1993). Further, the fungal treatment of the extraction-stage effluent from chlorine bleaching of kraft pulp with *Rhizopus oryzae* removed 100% of 5-chloro-, and 55% of 4,5-dichloroguaiacol (Nagarathnamma and Bajpai, 1999). For the interested reader, a table summarizing more reports about the removal of chlorinated guaiacols is provided in the Supplementary Material (Table S1).

Besides their undesired occurrence, chlorinated guaiacol derivatives may also exert positive effects: at least 3-chloroguaiacol showed antifungal activity against the fungus *Trichophyton mentagrophytes*, a dermatophyte (Pinto et al., 2001).

In the physiological context, 4-iodoguaiacol has been reported as metabolization product formed from 4-iodoanisole by rat liver microsomes. Accordingly, halogenated aromatic compounds can be further processed by the human body, so that the physiological derivatives also need to be regarded when it comes to a rating of their potential physiological effects *in vivo*. Besides this iodinated guaiacol and the mentioned chlorinated compounds, no other halogenated derivatives have been reported until today, let alone in the context of smell. However, previous studies of our group demonstrated that halogenated aromatic compounds can be extremely odor potent, as was demonstrated for structurally related halogenated phenols (Strube et al., 2012).

In the present study, we expand our study on halogenated guaiacols, with systematic analysis of 14 compounds regarding their odor properties (odor thresholds and odor qualities), retention indices and mass spectrometric characteristics. The compiled analytical data together with the sensory information aid their future investigation, and support unveiling their potential olfactory and environmental impact.

## Materials and methods

### Chemicals

Chloroform, diethyl ether, diethyl ether absolute, methanol, tetrahydrofuran, 3-bromosalicylaldehyde, 3-chlorosalicyaldehyde, 2-iodophenol, 2-methoxyphenol (guaiacol), n-butyllithium (n-BuLi), 3,4-dihydro-2H-pyrane (DHP), anhydrous magnesium chloride, anhydrous magnesium sulfate, iodine, iodomethane, sodium sulfite, sodium bicarbonate, sodium bisulfite, sodium thiosulfate, potassium dihydrogenphosphate, p-formaldehyde, pyridinium p-toluenesulfonate (PPTS), hydrochloric acid conc., silver trifluoroacetate, tetrabutylammonium hydroxide, trimethylamine, trifluoroacetic anhydride and hydrogen peroxide were purchased from Sigma-Aldrich (Steinheim, Germany), chloroform D1 including TMS (0.03 vol%) and sodium hydroxide were purchased from Carl Roth (Karlsruhe, Germany), 2-methoxyphenyl acetate was purchased from TCI Chemicals (Zwijndrecht, Belgium) and silica gel (Normasil 60, 40–63 μm), potassium carbonate, sodium chloride, anhydrous sodium sulfate, dichloromethane, n-hexane and ethyl acetate were purchased from VWR International GmbH (Darmstadt, Germany). 4,5-Dichloro- and 5,6-dichloro-2-methoxyphenol were purchased from ABL Pharmatech LLC (Monmouth Junction, NJ, USA), 4-bromo-2-methoxyphenol and 2-bromo-6-methoxyphenol were purchased from abcr GmbH (Karlsruhe, Germany), 5-bromo- and 5-chloro-2-methoxyphenol from chemPUR (Karlsruhe, Germany), 2-chloro-6-methoxyphenol from OXCHEM (Irwindale, CA, USA), and 4-chloro- and 4-iodo-2-methoxyphenol from Sigma-Aldrich (Steinheim, Germany). All chemicals were used without further purification.

### Nuclear magnetic resonance (NMR) spectra

^1^H and ^13^C NMR spectra were recorded in CDCl_3_ on an Avance 360 spectrometer, 360 MHz, and Avance 600 spectrometer, 600 MHz (Bruker Biospin, Rheinstetten, Germany) at room temperature operated at 360 or 600 MHz (^1^H) and 90 or 150 MHz (^13^C), with tetramethylsilane (TMS) as internal standard.

### GC-FID, GC olfactrometry (GC-O), and GC-electron impact-mass spectrometry (GC-EI-MS)

GC-FID and GC-O analyses were performed with a Trace GC Ultra (Thermo Fisher Scientific GmbH, Dreieich, Germany) by using the following capillary columns: FFAP (30 m × 0.32 mm fused silica capillary, free fatty acid phase FFAP, 0.25 μm; Chrompack, Mühlheim, Germany) and DB5 (30 m × 0.32 mm fused silica capillary DB-5, 0.25 μm; J & W Scientific, Fisons Instruments, Ipswich, United Kingdom). For the sniffing experiments, the samples were applied by the on-column injection technique at 40°C. After 2 min, the temperature of the oven was raised at 40°C/min to 165°C, 10°C/min to 175°C, 40°C/min to 205°C, and 10°C/min to 240°C (FFAP), and held for 5 min. The flow rate of the carrier gas helium was 2.5 mL/min. At the end of the capillary, the effluent was split in a ratio 1:1 (by volume) into an FID and a sniffing port using two deactivated but uncoated fused silica capillaries (50 cm × 0.32 mm). The FID and the sniffing port were held at 250°C, respectively. GC-EI-MS analyses were performed with an Agilent MSD 5975C (Agilent Technologies, Waldbronn, Germany) and a Thermo ITQ 900 (Thermo Fisher Scientific, Dreieich, Germany) with the capillaries described above, using the same temperature programs as for the GC-FID and GC-O measurements. The samples were applied by the on-column injection technique at 40°C. After 2 min, the temperature of the oven was raised at 15°C/min to 280°C (DB5), or at 15°C/min to 240°C (FFAP), respectively, and held for 5 min. The flow rate of the carrier gas helium was 1.0 mL/min (MSD) or 2.5 mL/min (ITQ). Mass spectra in the electron impact mode (EI-MS) were generated at 70 eV.

### Retention indices (RI)

Retention indices were determined by the method previously described by Vandendool and Kratz by injection of n-alkanes solution C_6_ to C_26_ (Vandendool and Kratz, 1963).

### Panelists

Panelists were trained volunteers from the University of Erlangen (Erlangen, Germany), exhibiting no known illness at the time of examination and with audited olfactory function. In preceding weekly training sessions, the assessors were trained for at least half a year in recognizing orthonasally about 150 selected known odorants at different concentrations according to their odor qualities, and in naming these according to an in-house developed flavor language. Furthermore, the panel was trained every 2 weeks on specific attributes with the help of specifically developed smell sticks; in the course of this training, all panelists also had to fill the same questionnaire (hedonic, intensity) to obtain insights into their specific sensitivities or insensitivities which were systematically recorded. Based on these tests, panelists were tested regularly if they comply with the established flavor language.

### Odor threshold values

Thresholds in air were determined by means of GC-O with (*E*)-2-decenal as internal standard on one of the columns given above (Boelens and Van Gemert, 1986; Ullrich and Grosch, 1987; Czerny et al., 2011). Of every dilution, 2 μL were injected into the GC system. In total, the thresholds were determined by 5 panelists (2 males, 3 females), with each experiment being conducted once. Every compound was orthonasally analyzed. GC analyses were performed on capillary DB5 and FFAP as already described. The purity of all commercial available and synthesized compounds was taken into account in the GC-O experiments. All synthesized compounds were further checked for potential odorous impurities by sniffing each single substance on both capillaries of different polarity, to exclude potential interferences.

### Odor quality determination

The odor qualities, determined during GC-O evaluation, were related to odor qualities of commercially available reference compounds. Panelists were asked to freely choose the respective odor quality descriptors based on the in-house developed flavor language (cf. chapter 2.5 Panelists). No additional descriptors were supplied to the panelists. The panelists determined the qualities during sniffing of the solution corresponding to FD 1 (injection of 2 μL). Additionally, the panelists were instructed to record any changes in odor qualities in all following dilutions. Testing was carried out according to an in-house developed protocol for minimization of substance hazard, and subjecting just minimum levels of odorants to gas chromatographic-olfactometric evaluation.

### Syntheses

All compounds were synthesized following literature procedures. If changes to the approach described in the literature have been made, they are described. Detailed synthetic prescriptions are provided in the Supplementary Material.

#### 3-chloro-2-methoxybenzaldehyde (Steen et al., 2009)

The product was given as a clear oily liquid. Yield: 496.8 mg (2.91 mmol, 51%). *M* = 170.59 g/mol. ^1^**H NMR** (600 MHz, CDCl_3_, rt): δ [ppm] = 4.02 (s, 3H, C**H**_3_), 7.20 (dt, *J* = 7.86 Hz, *J*′ = 7.86 Hz, *J*″ = 0.76 Hz, 1H, CHC**H**CH), 7.65 (dd, *J* = 7.80 Hz, *J*′ = 1.89 Hz, 1H, CHC**H**CCl), 7.76 (dd, *J* = 7.80 Hz, *J*′ = 1.89 Hz, 1H, HC = OC**H**CH), 10.39 (d, *J* = 1.13 Hz, 1H, **H**C = O). ^13^**C NMR** (90 MHz, CDCl_3_, rt): δ [ppm] = 189.0, 159.1, 136.4, 130.9, 128.8, 127.0, 125.2, 63.2. **MS-EI**, *m/z* (relative intensity in%): 170 (88, M), 169 (39, M^+^), 155 (74), 154 (64), 152 (100), 110 (41), 99 (60), 77 (44), 75 (53), 63 (58).

#### 3-chloroguaiacol (3-chloro-2-methoxyphenol, entry 1) (Steen et al., 2009)

The product was given as a clear oily liquid. Yield: 351.6 mg (2.22 mmol, 79%). *M* = 158.58 g/mol. Purity (GC): 100%. ^1^**H NMR** (600 MHz, CDCl_3_, rt): δ [ppm] = 3.93 (s, 3H, C**H**_3_), 5.72 (br. s, 1H, O**H**), 6.86–6.97 (m, 3H, C**H**C**H**C**H**). ^13^**C NMR** (90 MHz, CDCl_3_, rt): δ [ppm] = 150.1, 143.4, 126.9, 125.1, 121.7, 114.2, 61.1. **MS-EI**, *m/z* (relative intensity in %): 160 (22, M^−^), 158 (68, M^+^), 145 (33), 143 (100), 117 (16), 115 (48), 107 (8), 79 (13), 63 (9), 51 (26).

#### 3-bromo-2-methoxybenzaldehyde (Erickson et al., 2012)

The product was given as a yellowish oily liquid. Yield: 747.2 mg (3.47 mmol, 79%). *M* = 215.04 g/mol. ^1^**H NMR** (600 MHz, CDCl_3_, rt): δ [ppm] = 4.00 (s, 3H, C**H**_3_), 7.14 (dt, *J* = 8.03 Hz, *J*′ = 8.03 Hz, *J*″ = 0.76 Hz, 1H, CHC**H**CH), 7.81 (dd, *J* = 4.14 Hz, *J*′ = 1.51 Hz, 1H, HC = OCC**H**CH), 7.82 (dd, *J* = 4.14 Hz, *J*′ = 1.51 Hz, 1H, CHC**H**CBr), 10.37 (d, *J* = 1.13 Hz, 1H, **H**C = O). ^13^**C NMR** (90 MHz, CDCl_3_, rt): δ [ppm] = 188.6, 159.8, 139.0, 130.5, 127.4, 125.3, 117.7, 63.0. **MS-EI**, *m/z* (relative intensity in %): 216 (79, M^−^), 214 (76, M^+^), 200 (40), 199 (74), 198 (100), 196 (85), 170 (37), 77 (44), 75 (38), 63 (49).

#### 3-bromoguaiacol (3-bromo-2-methoxyphenol, entry 7) (Erickson et al., 2012)

The product was given as a yellow oily liquid. Yield: 539.4 mg (2.66 mmol, 79%). *M* = 203.04 g/mol. Purity (GC): 100%. ^1^**H NMR** (600 MHz, CDCl_3_, rt): δ [ppm] = 3.92 (s, 3H, C**H**_3_), 5.73 (br. s, 1H, O**H**), 6.91 (m, 2H, COHC**H**C**H**CH), 7.06 (dd, *J* = 7.09 Hz, *J*′ = 2.50 Hz, 1H, CHC**H**CBr). ^13^**C NMR** (90 MHz, CDCl_3_, rt): δ [ppm] = 150.1, 144.6, 125.9, 124.7, 115.8, 115.0, 61.1. **MS-EI**, *m/z* (relative intensity in %): 204 (94, M^−^), 202 (96, M^+^), 189 (100), 187 (99), 161 (37), 159 (38), 107 (22), 79 (21), 63 (14), 51 (34).

#### 3-iodosalicylaldehyde (Brady et al., 2012)

The product was given as yellow needles. Yield: 855.2 mg (3.45 mmol, 38%). *M* = 248.02 g/mol. ^1^**H NMR** (600 MHz, CDCl_3_, rt): δ [ppm] = 6.85 (t, *J* = 7.67 Hz, 1H, CHC**H**CH), 7.58 (dd, *J* = 7.66 Hz, *J*′ = 1.59 Hz, 1H, HC = OC**H**CHCH), 8.01 (dd, *J* = 7.73 Hz, *J*′ = 0.45 Hz, 1H, CHC**H**CI), 9.78 (s, 1H, **H**C = O), 11.83 (br. S, 1H, O**H**). ^13^**C NMR** (DEPTQ, 150 MHz, CDCl_3_, rt): δ [ppm] = 195.9, 160.4, 146.1, 134.0, 121.6, 120.6, 85.5. **MS-EI**, *m/z* (relative intensity in %): 249 (9, M^−^), 248 (100, M), 247 (45, M^+^), 120 (6), 103 (3), 92 (10), 75 (5), 64 (4), 63 (8), 62 (4).

#### 3-iodo-2-methoxybenzaldehyde, according to Erickson et al. (2012)

The educt used in the given literature, 3-bromosalicylaldehyde, was replaced by 3-iodosalicylaldehyde.

The product was given as a beige oily liquid. Yield: 640.6 mg (2.44 mmol, 71%). *M* = 262.05 g/mol. ^1^**H NMR** (600 MHz, CDCl_3_, rt): δ [ppm] = 4.96 (s, 3H, C**H**_3_), 7.02 (dt, *J* = 7.75 Hz, *J*′ = 7.75 Hz, *J*″ = 0.76 Hz, 1H, CHC**H**CH), 7.83 (dd, *J* = 7.77 Hz, *J*′ = 1.70 Hz, 1H, HC = OCC**H**CH), 8.04 (dd, *J* = 7.82 Hz, *J*′ = 1.70 Hz, 1H, CHC**H**CI), 10.33 (d, *J* = 0.76 Hz, 1H, **H**C = O). ^13^**C NMR** (DEPTQ, 150 MHz, CDCl_3_, rt): δ [ppm] = 189.3, 162.7, 145.5, 130.3, 129.1, 126.5, 92.9, 64.0. **MS-EI**, *m/z* (relative intensity in %): 262 (48, M), 261 (12, M^+^), 247 (16), 245 (23), 244 (100), 220 (18), 216 (21), 118 (11), 75 (10), 63 (14).

#### 3-iodoguaiacol (3-iodo-2-methoxyphenol, entry 11), according to Erickson et al. (2012)

The educt used in the given literature, 3-bromo-2-methoxybenzaldehyde, was replaced by 3-iodo-2-methoxybenzaldehyde.

The product was given as a yellowish oily liquid. Yield: 439.8 mg (1.76 mmol, 84%). *M* = 250.03 g/mol. Purity (GC): 89.2%. ^1^**H NMR** (600 MHz, CDCl_3_, rt): δ [ppm] = 3.88 (s, 3H, C**H**_3_), 5.71 (br. s, 1H, O**H**), 6.78 (t, 2H, CHC**H**CH), 6.95 (dd, *J* = 8.18 Hz, *J*′ = 1.51 Hz, 1H, HC = OC**H**CH) 7.30 (dd, *J* = 7.94 Hz, *J*′ = 1.51 Hz, 1H, CHC**H**CI). ^13^**C NMR** (DEPTQ, 150 MHz, CDCl_3_, rt): δ [ppm] = 149.2, 147.2, 130.6, 126.9, 116.2, 89.7, 61.1. **MS-EI**, *m/z* (relative intensity in %): 251 (8, M^−^), 250 (100, M^+^), 235 (55), 207 (13), 108 (6), 107 (6), 79 (4), 65 (5), 52 (4), 51 (5).

#### 5-iodo-2-methoxyphenylacetate (Banwell et al., 2006)

The product was given as a yellow solid. Yield: 2.48 g (8.49 mmol, 84%). *M* = 292.07 g/mol. **MS-EI**, *m/z* (relative intensity in %): 292 (15, M^+^), 251 (8), 250 (100), 235 (42), 207 (9), 123 (4), 79 (9), 51 (8), 50 (3), 43 (10).

#### 5-iodoguaiacol (5-iodo-2-methoxyphenol, entry 13) (Banwell et al., 2006)

The product was given as a beige solid. Yield: 615.2 mg (2.46 mmol, 29%). *M* = 250.04 g/mol. Purity (GC): 96.9%. ^1^**H-NMR** (600 MHz, CDCl_3_, rt): δ [ppm] = 3.88 (s, 3H, CH_3_), 5.59 (br. s, 1H, OH), 6.61 (d, *J* = 8.48 Hz, 1H, CHC**H**COCH_3_), 7.17 (dd, *J* = 8.48 Hz, *J*′ = 2.04 Hz, 1H, C**H**CHCOCH_3_), 7.24 (d, *J* = 2.04 Hz, 1H, **6**). ^13^**C-NMR** (DEPTQ, 150 MHz, CDCl_3_, rt): δ [ppm] = 146.5, 146.4, 129.0, 123.4, 112.5, 83.0, 6.0. **MS-EI**, *m/z* (relative intensity in %): 251 (8, M^−^), 250 (100, M^+^), 236 (4), 235 (53), 207 (23), 108 (1), 62 (2), 52 (3), 51 (3), 50 (1).

#### 2-(2-methoxyphenoxy)oxane, according to Weinstabl et al. (2013)

The literature procedure was slightly modified. After 4 h stirring at room temperature (like it is carried out in literature), another 29.2 mL DHP and 80 mg PPTS were added additionally, and the reaction mixture was stirred for another 72 h.

The product was given as a beige, oily liquid. Yield: 1.98 g (9.51 mmol, 59%). *M* = 208.26 g/mol. ^1^**H-NMR** (600 MHz, CDCl_3_, rt): δ [ppm] = 1.67 (m, 3H, OCH_2_C**H**_2_C**H**_2_), 1.93 (m, 2H, C**H**_2_C**H**_2_CHO), 2.07 (m, 1H, C**H**_2_CHO), 3.61 (m, 1H, C**H**_2_OCH), 3.87 (s, 3H, CH_3_), 4.03 (m, 1H, C**H**_2_OCH), 5.40 (t, *J* = 3.40 Hz, 1H, OC**H**O), 6.95 (m, 3H, C**H**C**H**C**H**COCH_3_), 7.15 (dd, *J* = 7.98 Hz, *J*′ = 1.51 Hz, 1H, CHC**H**CO). **MS-EI**, *m/z* (relative intensity in %): 125 (8), 124 (100), 109 (32), 85 (20), 84 (5), 81 (12), 67 (7), 57 (6), 55 (7), 41 (5).

#### 6-iodoguaiacol (2-iodo-6-methoxyphenol, entry 14) (Weinstabl et al., 2013)

The product was given as yellow solid. Yield: 430.7 mg (1.72 mmol, 35%). *M* = 250.04 g/mol. Purity (GC): 95.5%. ^1^**H-NMR** (600 MHz, CDCl_3_, rt): δ [ppm] = 3.90 (s, 3H, CH_3_), 6.10 (br. s, 1H, OH), 6.64 (t, *J* = 7.96 Hz, 1H, ICCHC**H**), 6.84 (dd, *J* = 8.01 Hz, *J*′ = 1.31 Hz, 1H, CHC**H**COCH_3_), 7.30 (dd, *J* = 8.01 Hz, *J*′ = 1.31 Hz, 1H, ICC**H**CH). ^13^**C-NMR** (DEPTQ, 150 MHz, CDCl_3_, rt): δ [ppm] = 146.2, 145.7, 130.6, 121.6, 110.7, 81.4, 56.2. **MS-EI**, *m/z* (relative intensity in %): 251 (8, M^−^), 250 (100, M^+^), 235 (48), 207 (12), 108 (11), 107 (7), 79 (6), 53 (5), 52 (7), 51 (7).

## Results

In total, five halogenated guaiacol derivatives, substituted in position 3 (meta), 5 (meta), and 6 (ortho), were successfully synthesized in the current study. All compounds were successfully generated following common procedures. GC-O and GC-MS analyses demonstrated high purities of the 3-chloro-, 3-bromo-compounds of 100% in each case. 3-Iodoguaiacol could be obtained with a purity of 89%, 5-iodoguaiacol with 97%, and 6-iodoguaiacol with 96%.

**Figure 1:**
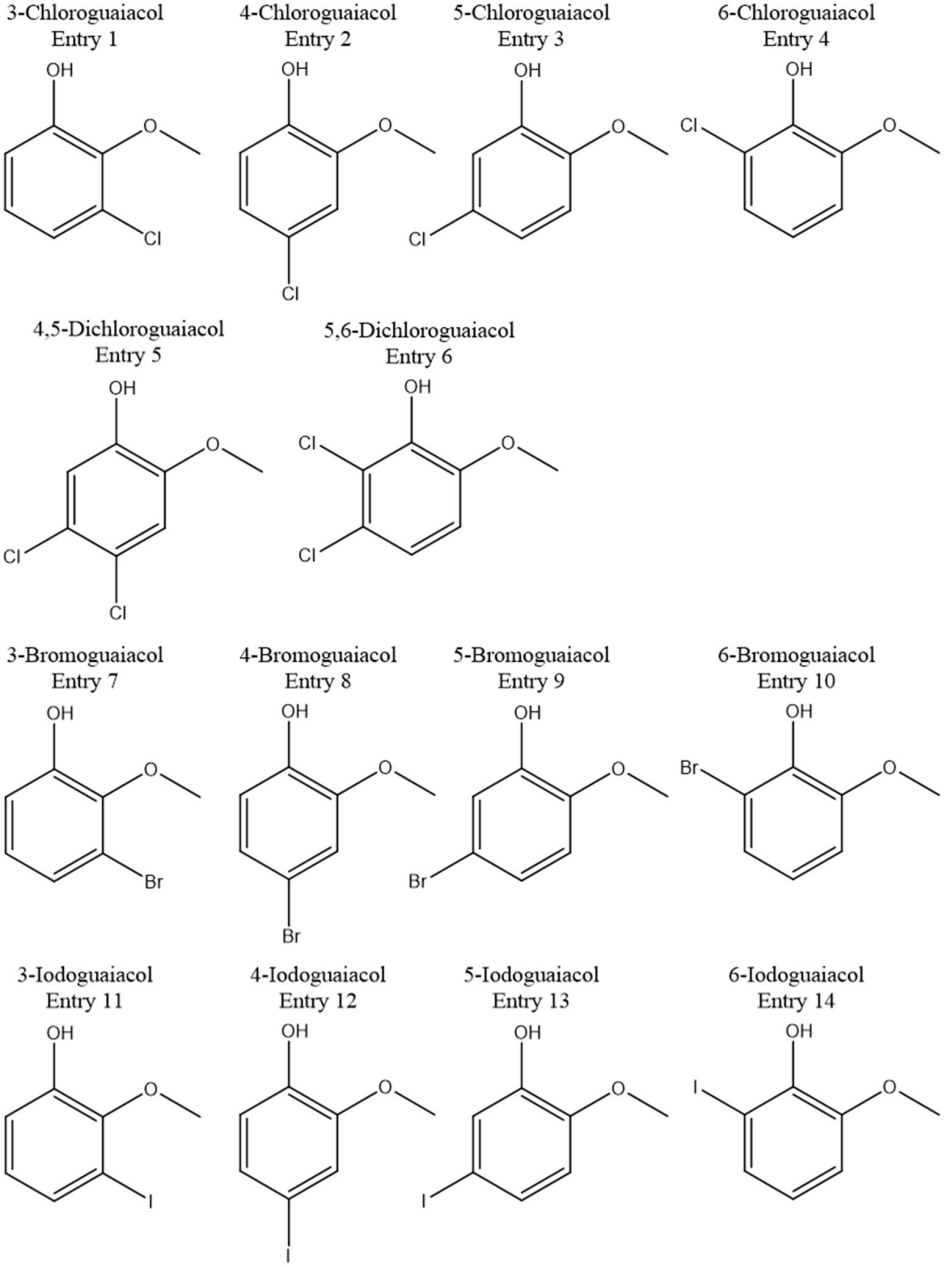
Summary of all investigated compounds.

**Figure 2:**
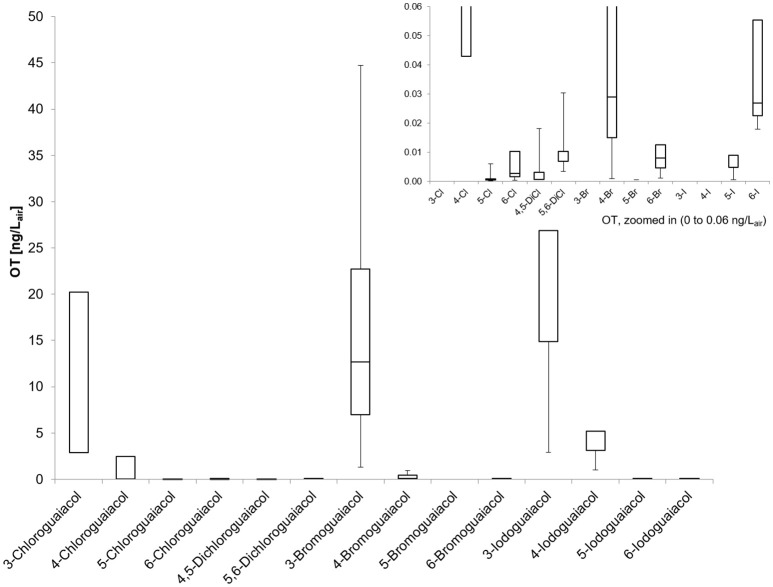
Boxplot of the odor thresholds for all investigated halogenated guaiacol derivatives (five panelists, two males, three females). Mean value (± *SD*), markers at minimum and maximum OT, box perc. 25–75%; Data presented as ranges with the respective whiskers (upper as well as lower bar are representing the highest and the lowest measured odor threshold values, cf. Table 2).

Accordingly, the odor threshold values as well as the odor qualities of these synthesized and the commercially available derivatives (mono-substituted in position3 (meta), 4 (para), 5 (meta), 6 (ortho), and di-substituted in positions 4 and 5, or 5 and 6 in relation to the hydroxyl function) could be determined by means of GC-O analysis in the next step. A summary of the structures of all investigated compounds is given in Figure 1. In Table 1 and Figure 2, the median odor threshold values in air are given for all investigated substances together with the respective odor threshold ranges, the factors between the highest and the lowest individual odor threshold value, and their odor qualities. The single values of each individual panelist and the geometric means of the odor threshold values are additionally listed in Table 2, for the sake of completeness. As in our previous publications, however, mainly the median and single values will be discussed as they appear to be more representative with regard to the panel size, because strong deviations between the single values would influence the geometric mean more significantly than the median (Lorber and Buettner, 2015; Lorber et al., 2016; Schranz et al., 2017).

**Table 1:** Retention indices (RI), odor thresholds (OT, median, range, and factor between highest and lowest OT) and odor qualities of all investigated halogenated guaiacol derivatives.

**Odorant**	**RI^a^**		**OT [ng/L**_**air**_**]^b^**			**Odor qualities^c^^,^^d^**
	**DB5**	**FFAP**	**Median**	**Range**	**Factor (high/low)**	
3-Chloroguaiacol	1197	2108	5.7	2.9–23	8	*smoky*, medical
4-Chloroguaiacol	1290	2213	0.35	0.043–2.8	65	*sweet*, vanilla-like
5-Chloroguaiacol	1298	2246	0.00072	0.00018–0.0058	49	*smoked*, smoky, ham-like
6-Chloroguaiacol	1322	2250	0.00251	0.000313–0.010022	32	*smoky*, sweet
4,5-Dichloroguaiacol	1518	2610	0.0025	0.00062–0.02	32	smoky, sweet, vanilla-like
5,6-Dichloroguaiacol	1550	2645	0.0068	0.0034–0.027	8	smoky, medical, plaster-like
3-Bromoguaiacol	1292	2272	11	1.3–43	33	musty, old
4-Bromoguaiacol	1379	2370	0.029	0.00092–0.94	1022	*vanilla-like, sweet*, smoky
5-Bromoguaiacol	1397	2420	0.0023	0.00057–0.018	32	*smoky*, sweet
6-Bromoguaiacol	1423	2415	0.0046	0.0012–0.0092	8	medical, smoky, plaster-, plastic-like
3-Iodoguaiacol	1409	2537	23	2.9–46	16	*musty*, moldy
4-Iodoguaiacol	1510	2564	4.1	1.0–16	16	*vanilla-like*, smoky, sweet
5-Iodoguaiacol	1519	2631	0.0048	0.0006–2.5	4167	*sweet*, smoked
6-Iodoguaiacol	1537	2616	0.036	0.018–0.15	8	medical

a *Retention indices were determined as described by Vandendool and Kratz (1963)*.

b *Odor thresholds in air were determined as described by Ullrich and Grosch (1987)*.

c *Odor qualities as perceived at the sniffing port*.

d *Underlined attributes are the main odor qualities. They were named by the majority of the panel*.

**Table 2:** Odor thresholds OT (GC-O) of all 5 panelists of all investigated halogenated guaiacol derivatives.

**Odorant**	**OT in air (ng/L)^a^^,^^b^**					
	**Geometric mean**	**P 1**	**P 2**	**P 3**	**P 4**	**P 5**
3-Chloroguaiacol	8.6	5.7	5.7	23	23	2.9
4-Chloroguaiacol	0.53	0.35	0.35	2.8	0.043	2.8
5-Chloroguaiacol	0.00072	0.0058	0.00036	0.00072	0.00018	0.00072
6-Chloroguaiacol	0.00251	0.01002	0.00251	0.01002	0.001253	0.000313
4,5-Dichloroguaiacol	0.0033	0.02	0.0025	0.0025	0.00062	0.005
5,6-Dichloroguaiacol	0.0068	0.027	0.0034	0.0068	0.0068	0.0034
3-Bromoguaiacol	9.3	5.3	1.3	11	43	21
4-Bromoguaiacol	0.044	0.47	0.00092	0.029	0.015	0.94
5-Bromoguaiacol	0.0026	0.018	0.0023	0.0023	0.00057	0.0023
6-Bromoguaiacol	0.0035	0.0092	0.0092	0.0046	0.0012	0.0012
3-Iodoguaiacol	15	2.9	11	23	46	23
4-Iodoguaiacol	3.5	1.0	4.1	4.1	2.0	16
5-Iodoguaiacol	0.0073	0.0048	0.0006	2.5	0.0006	0.0048
6-Iodoguaiacol	0.043	0.15	0.036	n.p.	0.018	0.036

a *Odor thresholds in air were determined as described by Ullrich and Grosch (1987)*.

b *n.p.: Panelist was unable to perceive the odorant*.

Regarding the odor qualities (Table 1), the investigated halogenated guaiacol derivatives were generally described as *smoky, sweet, vanilla-like, medicinal* and *plaster-like*. 3-Chloro-, 5-chloro-, 6-chloro-, 4,5-dichloro-, 5,6-dichloro-, and 5-bromoguaiacol primarily elicited *smoky* odor impressions, often accompanied by *vanilla-like* and *sweet* notes. 4-Chloro-, 4-bromo-, 4-iodo-, and 5-iodoguaiacol ostensibly smelled *sweet* and *vanilla-like*. 3-Bromo- and 3-iodoguaiacol were described as *musty*. A *medicinal* smell was reported by the panelists for 6-bromo- and 6-iodoguaiacol. In most cases, compounds were reported by the panelists to evoke two to four odor qualities. This led, on average, to an equalized weighting of the attributes' allocation. For instance, 3-chloroguaiacol was described as *smoky* and *medicinal*, while 6-chloroguaiacol primarily elicited a *smoky* odor impression, followed by *sweet* in second place. The smell of 4,5-dichloroguaiacol was found to be a combination of *smoky, sweet* and *vanilla-like*; the smell of 5,6-dichloroguaiacol was composed of *smoky, medicinal* and *plaster-like* impressions. The named attributes for 6-bromoguaiacol were *medicinal, smoky, plaster-* and *plastic-like* with equal weighting, while 6-iodoguaiacol was exclusively described as *medicinal*.

Altogether, very low odor thresholds were determined for all investigated compounds (Table 1). The lowest median odor threshold was found for 5-chloroguaiacol with a value of 0.00072 ng/L_air_, which is in a comparative range as the lowest value determined in the study of the alkylated, alkenylated and methoxylated guaiacol derivatives, namely the very low odor threshold of 5-methoxyguaiacol with 0.00018 ng/L_air_. In comparison to guaiacol itself, with a median odor threshold of 0.084 ng/L_air_ (Schranz et al., 2017) and therefore being already a quite potent substance itself, the odor threshold of 5-chloroguaiacol is nearly a factor of 120 lower than that of the parent structure. In contrast to that, the median odor thresholds of 6-chloro-, 4,5-dichloro-, 5,6-dichloro-, 5-bromo-, 6-bromo-, and 5-iodoguaiacol were represented values between 0.0025 and 0.0068 ng/L_air_, and, accordingly, ranged between those of guaiacol and 5-chloroguaiacol. Higher values were recorded for 4-bromo- and 6-iodoguaiacol with 0.029 and 0.036 ng/L_air_, respectively, and 4-chloroguaiacol with 0.35 ng/L_air_. Thresholds with 5.7 and 4.1 ng/L_air_ were determined for 3-chloro- and 4-iodoguaiacol, whereas 3-bromo- and 3-iodoguaiacol showed the highest median odor thresholds of this study with 11 and 23 ng/L_air_.

Individual odor threshold values showed some variance, with factors between minimum and maximum individual odor threshold value commonly spanning values of 8–65. Only in two cases, extreme threshold variations were observed between panelists, namely a factor of 1022 for 3-bromoguaiacol and 4167 for 5-iodoguaiacol.

## Discussion

### Odor qualities

#### Main odor qualities

Overall, the following odor descriptors were named for all investigated compounds: *smoky, sweet, smoked, vanilla-like, musty*, and *medicinal*/*plaster-like* (Table 1). The main odor quality reported for basically all substances was *smoky* followed by *sweet, vanilla-like* and *medicinal*. With the sole exception of 4-chloroguaiacol, all chlorinated/dichlorinated derivatives were found to elicit predominantly *smoky*/*smoked* odor impressions, accompanied by *vanilla-like* and *sweet* notes, the latter two being the main odor qualities that were named for 4-chloroguaiacol. In case of the brominated guaiacols, 3-bromoguaiacol was the only compound described as *musty, old*, whereas 4-bromo- and 5-bromoguaiacol smelled *vanilla-like, sweet, smoky*, and *smoky, sweet*, respectively. Additionally, the attributes *medicinal, plaster-* and *plastic-like* were named for 6-bromoguaiacol. The same behavior regarding the named odor qualities was observed for the iodoguaiacols.

With regards to substitution patterns, it became evident that all compounds substituted in position 3 showed *smoky* and *medicinal* smells for the chlorinated derivatives, whereas the brominated and iodinated analogs elicited *musty, moldy* and *old* odor impressions. Para-substitution caused, in all cases, a *sweet* and *vanilla-like* smell, with an additional *smoky* note in case of 4-bromo- and 4-iodoguaiacol. While the panelists named the attributes *smoked, smoky*, and *ham-like* for 5-chlorguaiacol, 5-bromo-, and 5-iodoguaiacol showed a *sweet* scent resembling the *smoky* odor as is characteristic for guaiacol. The ortho-substituted compounds, however, displayed stronger deviations regarding their main odor descriptors. While 6-chloroguaiacol primarily smelled *smoky*, accompanied by a *sweet* impression, 6-bromoguaiacol elicited *medicinal, smoky, plaster-* and *plastic-like* odor notes. 6-Iodoguaiacol, on the other hand, was only described as *medicinal*. The two investigated dichlorinated derivatives only shared the attribute *smoky*. 4,5-Dichloroguaiacol was further described as *sweet* and *vanilla-like*, whereas 5,6-dichloroguaiacol showed *medicinal* and *plaster-like* impressions.

#### Individual odor qualities

Overall, the panelists were quite consistent regarding their reported odor qualities (Table S2, Supporting Information), whereas there were high variances between individual odor thresholds as will be discussed later on. Still, there were some exceptions for specific substances: panelist 6 exclusively described 3-chloroguaiacol as *vanilla-like*, whereas no other panelist named a *sweet* or *vanilla-like* note for this compound. While panelists 1, 2, and 3 found, besides the *smoky* odor, *sweet* and *vanilla-like* smells for 6-chloroguaiacol, panelist five noted *pool-, plaster-like* and *medicinal* odors, and panelist 6 perceived the substance as *artificial, plastic-like* and *pungent*. 5,6-Dichloroguaiacol elicited *plaster-like* or *medicinal* smells for panelists 2, 4, and 5. In contrast to this, panelist 1 and 3 named only one odor attribute for this compound, namely *smoky*. Two panelists described 4-bromoguaiacol as *vanilla-like* only, whereas the same substance was perceived as *smoky* or *musty* odors by the rest of the panel. Moreover, four out of five panelists perceived 3-iodoguaiacol as *musty* and *moldy*, whereas only panelist 1 described this substance as *smoky*. In case of 6-iodoguaiacol, a clear anosmia could be observed, as panelist 3 was not able to perceive this compound. Panelist 1 and 2 named only one attribute for 6-iodoguaiacol, *smoked* or *smoky*, respectively, while *medicinal* and *plaster-* or *plastic-like* smells were perceived by panelist 5 and 6.

Accordingly, a direct correlation of specific smell impressions with specific patterns of substitution is hard to establish, especially when considering the variable perceptual effects that were observed between different individuals. In this context, it is interesting to note that the variable attribute naming is presumably no linguistic effect, as panelists stated clear smell correlations with specific substances that they were trained on, for example vanillin or guaiacol. That means that smell naming was rather linked to olfactory references, rather revealing that the individuals perceived different compounds as smelling comparable, indicating a factual difference in smell perception rather than an inconsistency in naming. In our opinion, such smell cross-referencing will need to become much more important in future structure-odor investigations as smell referencing to other substances for the purpose of comparison will surely help ruling out the potential of linguistic bias, and will support inter-laboratory comparison of smell perception.

### Odor thresholds in air

Regarding the odor threshold values, we observed that the individual values of the five panelists for some substances varied within relatively broad ranges. As already mentioned, we discuss the median odor thresholds in the following (Tables 1, 2).

#### Median odor thresholds

Compounds substituted in position 3 (meta) in relation to the hydroxy function showed the highest median odor threshold values, the lowest were observed for the compounds substituted in position 5, whereas the values of the para- and ortho-substituted derivatives ranged between those of the position 3- and 5-substituted analogs. Thereby, the thresholds of the 5-monohalogenated derivatives were lower by factors as high as about 4,800–8,000 compared to the corresponding compounds substituted in position 3, comprising up to four orders of magnitude between values (Table 1), suggesting that these compounds are either exceptionally prone to a specific receptor interaction. Apart from that, compounds substituted in ortho-position also showed very low odor thresholds, 640- to even 2,400-fold lower values in comparison to substances halogenated in position 3. Para-substituted derivatives, on the other hand, showed only moderately lower threshold values in comparison to those of compounds substituted in position 3, being lower by factors between 6 and 380.

When regarding the three substituents chlorine, bromine and iodine specifically, we found that the chloroguaiacols showed the lowest median odor thresholds ranging between 5.7 ng/L_air_ for 3-chloroguaiacol and 0.00072 ng/L_air_ for 5-chloroguaiacol (lowest odor threshold determined in this study), followed by the bromoguaiacols with values ranging between 11 ng/L_air_ for 3-bromoguaiacol and 0.0023 ng/L_air_ for 5-bromoguaiacol, and values of 23 and 0.0048 ng/L_air_ for 3-iodoguaiacol and 5-iodoguaiacol, respectively, representing an increase in threshold level in each case. This increase is likely to be caused by the strong increase in molar mass from chloro- to iodo-derivatives.

With regards to the two di-chlorinated compounds, extremely low odor thresholds levels were also observed with 0.0025 ng/L_air_ for 4,5-dichloroguaiacol and 0.0068 ng/L_air_ for 5,6-dichloroguaiacol, comparable with the values of the following mono-substituted compounds: 6-chloro- (0.0025 ng/L_air_), 5-bromo- (0.0023 ng/L_air_), 6-bromo- (0.0046 ng/L_air_), and 5-iodoguaiacol (0.0048 ng/L_air_). This is, to some extent, surprising as the molecular weight of the di-chlorinated compounds is relatively high, yet, their high odor potency is on the other hand well in line with very low odor threshold levels that have previously been reported for other multi-halogenated compounds such as trichloroanisols (Strube and Buettner, 2010). However, this cannot be regarded as a general rule, and other di-halogenated derivatives would further need to be analyzed to get a comprehensive picture of the impact of multi-halogenation.

Finally, one might speculate if the very low odor threshold of 5-chloroguaiacol may indicate a specific biological relevance of this compound, irrespective of the fact that it has not yet been reported as a substance in nature. In this respect, the extremely low odor threshold of 0.00072 ng/L_air_ and, as a consequence thereof, the high smell potency of the substance together with its likely appearance, if any, at trace level, can be assumed to be the reason why this compound has not yet been reported as naturally occurring.

#### Individual odor thresholds

Regarding the individual values of all five panelists, the odor thresholds of the halogenated guaiacol derivatives showed variations between individuals within one to two orders of magnitude in the majority of cases. Thereby, the least variation was observed for 3-chloroguaiacol (2.9–23 ng/L_air_), 5,6-dichloroguaiacol (0.0034–0.027 ng/L_air_), and 6-bromoguaiacol (0.0012–0.0092 ng/L_air_). In contrast to this, a variation factor of as high as 1022 was recorded for 4-bromoguaiacol, with values ranging from 0.00092 to 0.94 ng/L_air_, thereby spanning three orders of magnitude. Apart from that, the highest deviation was observed for 5-iodoguaiacol with values ranging between 0.0006 and 2.5 ng/L_air_, which corresponds to a factor of 4167 or four orders of magnitude, respectively (cf. Table 1).

Furthermore, panelist 3 was unable to perceive 6-iodoguaiacol. In this context, it is interesting to note that the same panelist, was, however, able to smell the corresponding chlorinated and brominated derivatives, and with comparable sensitivity as the other panelists.

As already discussed in our previous studies, a series of factors might influence the perception of odorants (Lorber and Buettner, 2015; Lorber et al., 2016). Amongst others, it has been reported that the physiological or psychological state can modify the way an odorant is perceived: odor thresholds have been observed to increase after food intake whereas they can decrease in hunger state. Other influencing factors can be health, emotional state, gender, and, in case of women, pregnancy or menstrual cycle (Schmidt et al., 2010). Besides those physiological and psychological factors, genetic preposition in individual receptor repertoire but also differently expressed metabolizing enzyme systems at the periphery may cause inter-individual differences in odor perception (Mombaerts, 2001; Keller et al., 2007). Odorants have to pass the aqueous milieu of the nasal cavity before docking to the receptor proteins that are embedded in the cilia. Besides water binding glycoproteins, structure giving polypeptides and carbohydrates, this aqueous viscous colloidal mucus contains enzymes (e.g., members of the cytochrome P450 system) which are able to metabolize odorants before reaching the receptor sites, a process that is comprised within the term “peri-receptor events”; such biotransformation may impact the odor threshold and perceived odor quality of the respective odorants (Zhang et al., 2005; Chougnet et al., 2009; Nagashima and Touhara, 2010; Rehner and Daniel, 2010; Schilling et al., 2010; Lorber and Buettner, 2015). However, such biotransformation processes have not yet been reported for the investigated halogenated guaiacol derivatives. Accordingly, the question remains if the halogenated guaiacols are the molecular stimuli that directly activate the receptor sites.

## Conclusion

We found that all investigated halogenated guaiacol derivatives showed high odor potencies, which is reflected by their very low odor thresholds. Generally, the compounds elicited mostly pleasant odor impressions resembling those of guaiacol itself, namely *smoky, vanilla-like* and *sweet*. However, some compounds like 3-bromo-, 6-bromo-, 3-iodo-, and 6-iodoguaiacol were described as smelling unpleasant with *musty, moldy* and *medicinal* odor notes. Despite their low odor thresholds, none of the investigated substances has been previously identified in nature, only in wastewater of pulp and paper mills, and generally as contaminants. Due to the very low odor thresholds, especially of 5-chloroguaiacol with 0.00072 ng/Lair, it might be speculated, however, if there is any biological meaning of these halogenated guaiacol that developped through evolutionary processes, or if the halogen moiety itself renders the strong sensorial impact as non-intended side-effect. In any case, these questions may be better addressed in the future based on the analytical data (retention indices on two different columns, mass spectra, odor thresholds in air, and odor qualities) provided in this study, and may aid the future discovery and analysis of such halogenated derivatives.

## Author contributions

All authors listed have made a substantial, direct and intellectual contribution to the work, and approved it for publication.

### Conflict of interest statement

The authors declare that the research was conducted in the absence of any commercial or financial relationships that could be construed as a potential conflict of interest.
