# Supplementary material for: Influence of the Chemical Structure on Odor Qualities and Odor Thresholds of Halogenated Guaiacol-Derived Odorants
Source: Front Chem. 2017 Dec 18;5:120. doi: 10.3389/fchem.2017.00120 (PMC5741668; doi:10.3389/fchem.2017.00120)
Supplement: Supplementary file 1 [file DataSheet1.PDF]

Supporting Information

Influence of the chemical structure on odor qualities and odor thresholds of halogenated guaiacol-derived odorants

Florian Juhlke[a], Katja Lorber[a,b], Maria Wagenstaller[b], Andrea Buettner[a,b*]

[a]Professorship of Aroma Research, Department of Chemistry and Pharmacy, Emil Fischer Center, Friedrich-Alexander-Universität Erlangen-Nürnberg (FAU), Henkestr. 9, 91054 Erlangen, Germany
[b]Department of Sensory Analytics, Fraunhofer Institute for Process Engineering and Packaging IVV, Giggenhauser Str. 35, 85354 Freising, Germany

*Correspondence:

Andrea Buettner

andrea.buettner@ivv.fraunhofer.de

Synthesis

3-Chloro-2-methoxybenzaldehyde, Figure 1 (Steen et al., 2009)
NaOH pellets (763 mg, 19 mmol, 3.5eq) were dissolved in H_2O (20 mL). Tetrabutylammonium hydroxide solution (TBAH, 24 mL, ~1.5 M or ~40% in H_2O, ~36 mmol, ~ 6 eq.), CH_2Cl_2 (40 mL), and 3-chlorosalicylaldehyde (887 mg, 5.66 mmol, 1eq) were added. Iodomethane (1.8 mL, 4.1 g, 28.9 mmol, 5 eq.) was added dropwise, and the reaction mixture was stirred for 22 h at room temperature afterwards. The aqueous and organic layers were separated, and the aqueous layer was extracted three times with 50 mL CH_2Cl_2. The combined organic layers were dried over Na_2SO_4, and the solvent was removed under reduced pressure. The residue was purified by column chromatography (Silica gel; n-hexane/ethyl acetate = 5:1).

3-Chloroguaiacol (3-chloro-2-methoxyphenol, entry 1), Figure 1 (Steen et al., 2009)
Trifluoroacetic anhydride (3 mL, 4.5 g, 21.58 mmol, 8 eq.) was added dropwise to an ice-cold solution of H_2O_2 solution (493 mg, 30 wt.% in H_2O, 4.35 mmol, 1.6 eq.) and CH_2Cl_2 (2 mL), and stirred for 1 h at 0 °C. 3-Chloro-2-methoxybenzaldehyde (480 mg, 3.03 mmol, 1 eq.) and potassium dihydrogenphosphate (8 g, 58.78 mmol, 22 eq.) were dissolved in CH_2Cl_2 (20 mL) and cooled to 0 °C. The peroxide-solution was slowly added dropwise to the prepared 3-chloro-methoxybenzaldehyde solution. The reaction mixture was stirred for 2 h at 0°C. To stop the reaction, 10 mL brine and 10 mL 20% sodium bisulfite solution were added. After separation, the aqueous layer was extracted three times with 30 mL CH_2Cl_2. The combined organic layers were dried over Na_2SO_4, and the solvent was removed under reduced pressure. The residue was taken up in 15 mL MeOH, one drop HCl conc. was added, and the mixture left standing for 15 h at room temperature. After removal of the solvent, the crude product was purified by column chromatography (Silica gel; n-hexane/ethyl acetate = 4:1).

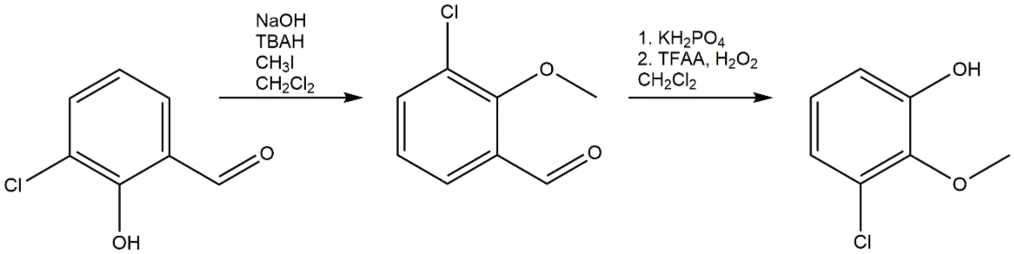

Figure S1: General synthetic route leading to 3-chloroguaiacol (entry 1)

3-Bromo-2-methoxybenzaldehyde, Figure 2 (Erickson et al., 2012)
NaOH pellets (595 mg, 14.88 mmol, 3.5 eq.) were dissolved in H_2O (20 mL). Tetrabutylammonium hydroxide solution (18 mL, ~1.5 M or ~40% in H_2O, ~27 mmol, ~ 6 eq.), CH_2Cl_2 (40 mL), and 3-bromosalicylaldehyde (888 mg, 4.42 mmol, 1 eq.) were added. Iodomethane (1.14 mL, 3.19 g, 22.49 mmol, 5 eq.) was added dropwise, and the reaction mixture was stirred for 18 h at room temperature afterwards. The aqueous and organic layers were separated, and the aqueous layer was extracted three times with 50 mL CH_2Cl_2. The combined organic layers were dried over Na_2SO_4, and the solvent was removed under reduced pressure. The residue was purified by column chromatography (Silica gel; n-hexane/ethyl acetate = 5:1).

3-Bromoguaiacol (3-bromo-2-methoxyphenol, entry 7), Figure 2 (Erickson et al., 2012)
Trifluoroacetic anhydride (3.8 mL, 5.74 g, 27.33 mmol, 8 eq.) was added dropwise to an ice-cold solution of H_2O_2 solution (606 mg, 30 wt% in H_2O, 5.35 mmol, 1.6 eq.) and CH_2Cl_2 (5 mL), and stirred for 1 h at 0 °C. 3-Bromo-2-methoxybenzaldehyde (721 mg, 3.35 mmol, 1 eq.) and potassium dihydrogenphosphate (10 g, 73.48 mmol, 22 eq.) were dissolved in 40 mL CH_2Cl_2 and cooled to 0 °C. The peroxide-solution was slowly added dropwise to the prepared 3-bromo-methoxybenaldehyde solution. The reaction mixture was stirred for 2 h at 0°C. To stop the reaction, 10 mL brine and 10 mL 20% sodium bisulfite solution were added. After separation, the aqueous layer was extracted three times with 30 mL CH_2Cl_2. The combined organic layers were dried over Na_2SO_4, and the solvent was removed under reduced pressure. The residue was taken up in 20 mL MeOH, one drop HCl conc. was added, and the mixture stirred for 15 h at room temperature. After removal of the solvent, the crude product was purified by column chromatography (Silica gel; n-hexane/ethyl acetate = 4:1).

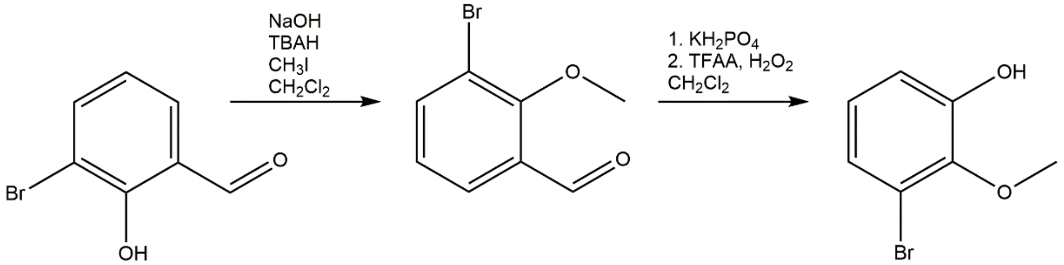

Figure S2: General synthetic route leading to 3-bromoguaiacol (entry 7)

3-Iodosalicylaldehyde, Figure 3 (Brady et al., 2012)
Under inert gas atmosphere, $MgCl_2$ (1.73 g, 18 mmol, 2 eq.), trimethylamine (TEA) (2.6 mL, 1.9 g, 19 mmol, 2 eq.), p-formaldehyde (810 mg, 27 mmol, 3 eq.) and 2-iodophenol (2 g, 9 mmol, 1 eq.) were dissolved in 60 mL dry THF. The reaction mixture was stirred under reflux for 16 h. After diluting with 100 mL Et_2O, the solution was washed three times with 50 mL HCl (2 N), once with 50 mL H_2O, and 50 mL brine. The organic layer was separated and dried over $MgSO_4$. After removal of the solvent under reduced pressure, the residue was twice recrystallized in EtOAc/n-hexane (1/1).

3-Iodo-2-methoxybenzaldehyde, Figure 3, according to (Erickson et al., 2012)
NaOH pellets (465 mg, 11.63 mmol, 3.5 eq.) were dissolved in H_2O (15 mL). Tetrabutylammonium hydroxide solution (15 mL, ~1.5 M or ~40% in H_2O, ~23 mmol, ~ 6 eq.), CH_2Cl_2 (30 mL), and 3-iodosalicylaldehyde (855 mg, 3.45 mmol, 1 eq.) were added. Iodomethane (1.1 mL, 2.5 g, 17.67 mmol, 5 eq.) was added dropwise, and the reaction mixture was stirred for 16 h at room temperature afterwards. The aqueous and organic layers were separated, and the aqueous layer was extracted three times with 50 mL CH_2Cl_2. The combined organic layers were dried over Na_2SO_4, and the solvent was removed under reduced pressure. The residue was purified by column chromatography (Silica gel; n-hexane/ethyl acetate = 5:1).

3-Iodoguaiacol (3-iodo-2-methoxyphenol, entry 11), Figure 3, according to (Erickson et al., 2012)
Trifluoroacetic anhydride (2.28 mL, 3.45 g, 16.4 mmol, 8 eq.) was added dropwise to an ice-cold solution of H_2O_2 solution (364 mg, 30 wt% in H_2O, 3.21 mmol, 1.6 eq.) and CH_2Cl_2 (5 mL), and stirred for 1 h at 0 °C. 3-Iodo-2-methoxybenzaldehyde (558 mg, 2.13 mmol, 1 eq.) and potassium dihydrogenphosphate (6 g, 44.1 mmol, 22 eq.) were dissolved in 25 mL CH_2Cl_2 and cooled to 0 °C. The peroxide-solution was slowly added dropwise to the prepared 3-bromo-methoxybenaldehyde solution. The reaction mixture was stirred for 2 h at 0°C. To stop the reaction, 10 mL brine and 10 mL 20% sodium bisulfite solution were added. After separation, the aqueous layer was extracted three times with 30 mL CH_2Cl_2. The combined organic layers were dried over Na_2SO_4, and the solvent was removed under reduced pressure. The residue was taken up in 15 mL MeOH, one drop HCl conc. was added, and the mixture stirred for 15 h at room temperature. After removal of the solvent, the crude product was purified by column chromatography (Silica gel; n-hexane/ethyl acetate = 4:1).

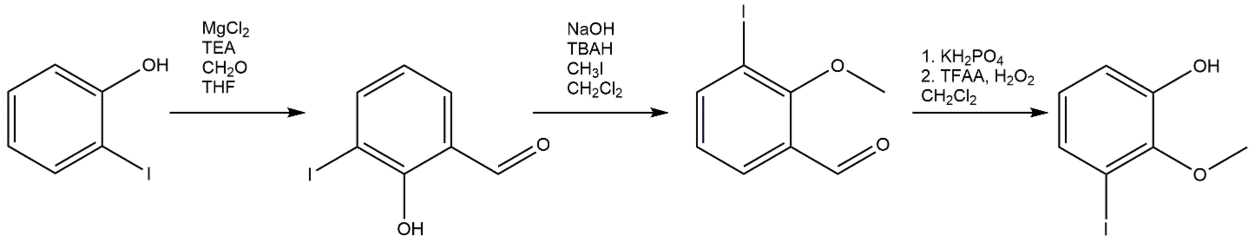

Figure S3: General synthetic route leading to 3-iodoguaiacol (entry 11)

5-Iodo-2-methoxyphenylacetate, Figure 4 (Banwell et al., 2006)
In a three necked flask equipped with a dropping funnel, 2-methoxyphenyl acetate (1.67 g, 10.05 mmol, 1 eq.) in chloroform (25 mL) maintained at 0 °C under inert gas atmosphere was treated, in one portion, with silver trifluoroacetate (2.89 g, 13.08 mmol, 1.3 eq.). To this mixture a solution of molecular iodine (2.56 g, 10.09 mmol, 1 eq.) in chloroform (62.5 mL) was added dropwise over 1 hour. The mixture was allowed to warm to room temperature during 1 h, stirred for another 4 h, washed with sodium thiosulfate (100 mL of a 20% w/v aqueous solution), and dried over $MgSO_4$.

5-Iodoguaiacol (5-iodo-2-methoxyphenol, entry 13), Figure 4 (Banwell et al., 2006)
A solution of 5-iodo-2-methoxyphenyl acetate (2.47 g, 8.46 mmol, 1 eq.) in methanol (70 mL) under inert gas atmosphere was treated in one portion with potassium carbonate (5.85 g, 42.33 mmol, 5 eq.). The mixture was stirred at room temperature for 16 h, and filtered through a sintered-glass funnel (once pore 4 and once pore 5). The solvent was removed under reduced pressure and the crude product was purified by column chromatography (Silica gel; n-hexane/ethyl acetate = 3:1).

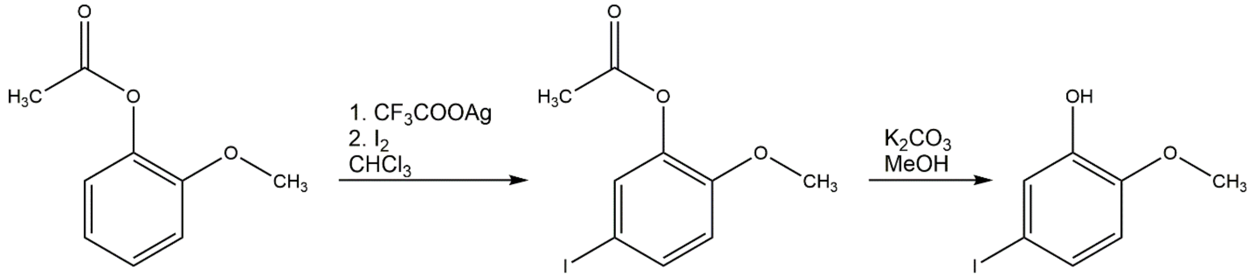

Figure S4: General synthetic route leading to 5-iodoguaiacol (entry 13)

2-(2-methoxyphenoxy)oxane, Figure 5, according to (Weinstabl et al., 2013)
To a solution of 2 g guaiacol (16.11 mmol, 1 eq.) in CH_2Cl_2 (14 mL) 3,4-dihydro-2H-pyran (DHP, 14.6 mL, 161.1 mmol, 10 eq.) and pyridinium *p*-toluenesulfonate (PPTS, 40 mg, 1.61 mmol, 0.1 eq.) were added. The reaction was stirred for 4 h at room temperature. The reaction progress was monitored by TLC. Another 29.2 mL DHP and 80 mg PPTS were added, and the reaction mixture was stirred for another 72 h. Saturated NaOH (12 mL) was added to stop the reaction. The mixture was extracted with CH_2Cl_2 and the combined organic layers were dried over $MgSO_4$. The solvent was removed under reduced pressure and the crude product was purified by column chromatography (Silica gel; n-hexane/ethyl acetate = 20:1).

6-Iodoguaiacol (2-iodo-6-methoxyphenol, entry 14), Figure 5 (Weinstabl et al., 2013)
Under inert gas atmosphere, to a solution of 2-(2-methoxyphenoxy)oxane (1.03 g, 4.95 mmol, 1.0 eq.) in THF (10 mL) n-BuLi (2.88 mL, 2.5 M in n-hexane, 7.2 mmol, 1.5 eq) was added slowly at 0 °C. The reaction was allowed to warm to room temperature and stirred under nitrogen atmosphere for 3 h. Subsequently, the mixture was cooled to -50 °C (methanol/liquid nitrogen bath) and iodine (2.44 g, 9.61 mmol, 2.0 eq.) dissolved in THF (15 mL) was added dropwise. After warming to room temperature overnight (16 h stirring) the reaction mixture was concentrated in vacuo and the residue was suspended in water and extracted three times with 30 mL CH_2Cl_2. The combined organic layers were washed with 50 mL saturated Na_2SO_3 solution and 50 mL H_2O. After drying over $MgSO_4$ and removal of the solvent under reduced pressure, the residue was adsorbed on silica, and purified by column chromatography (Silica gel; n-hexane/ethyl acetate = 3:1).

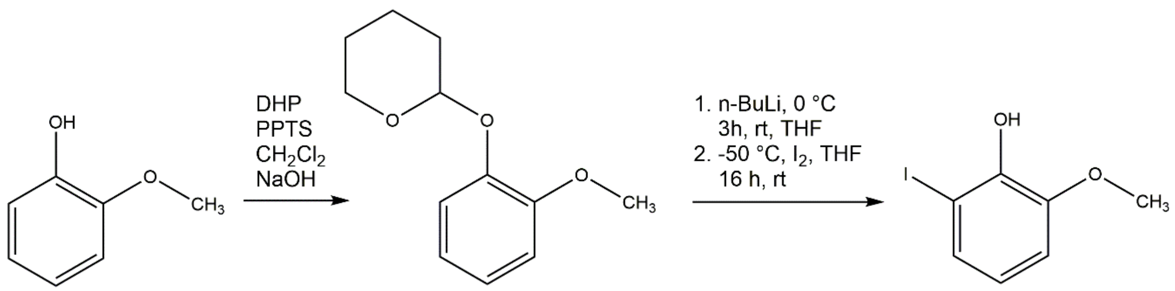

Figure S5: General synthetic route leading to 6-iodoguaiacol (entry 14).

Mass spectra

3-Chloroguaiacol (entry 1)

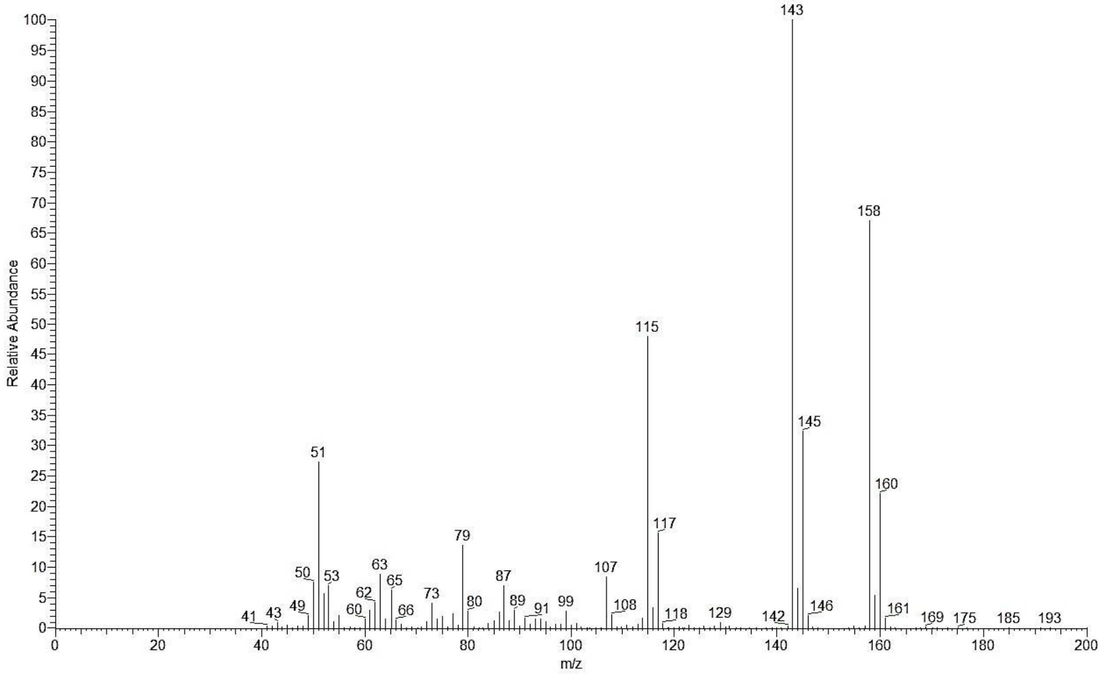

3-Bromoguaiacol (entry 7)

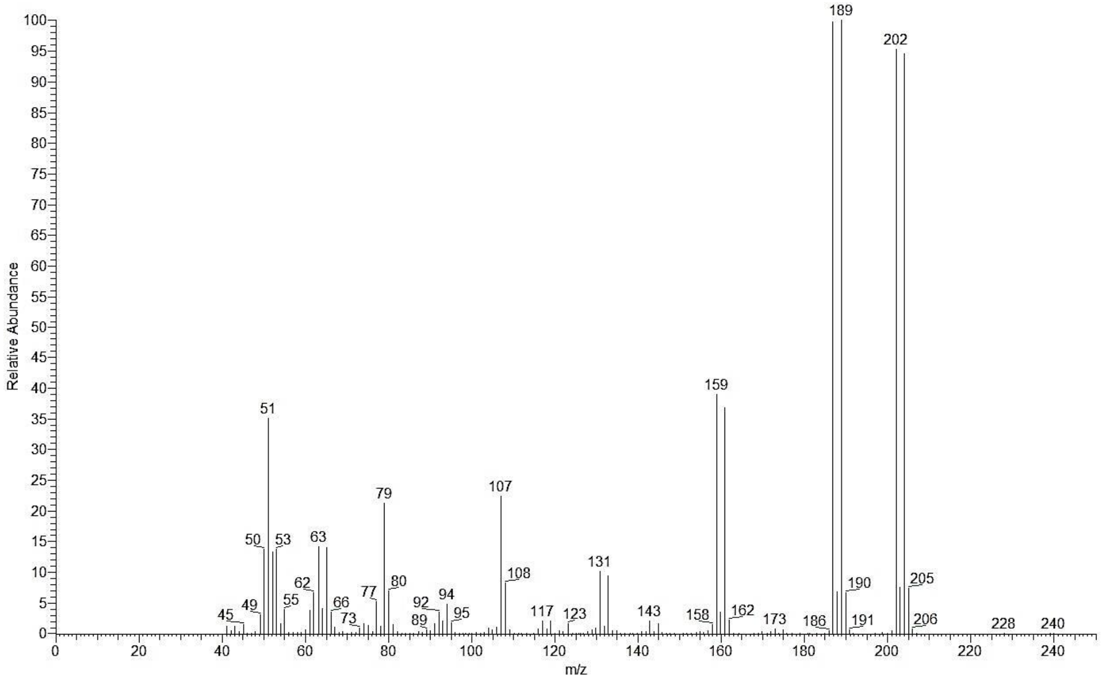

3-Iodoguaiacol (entry 11)

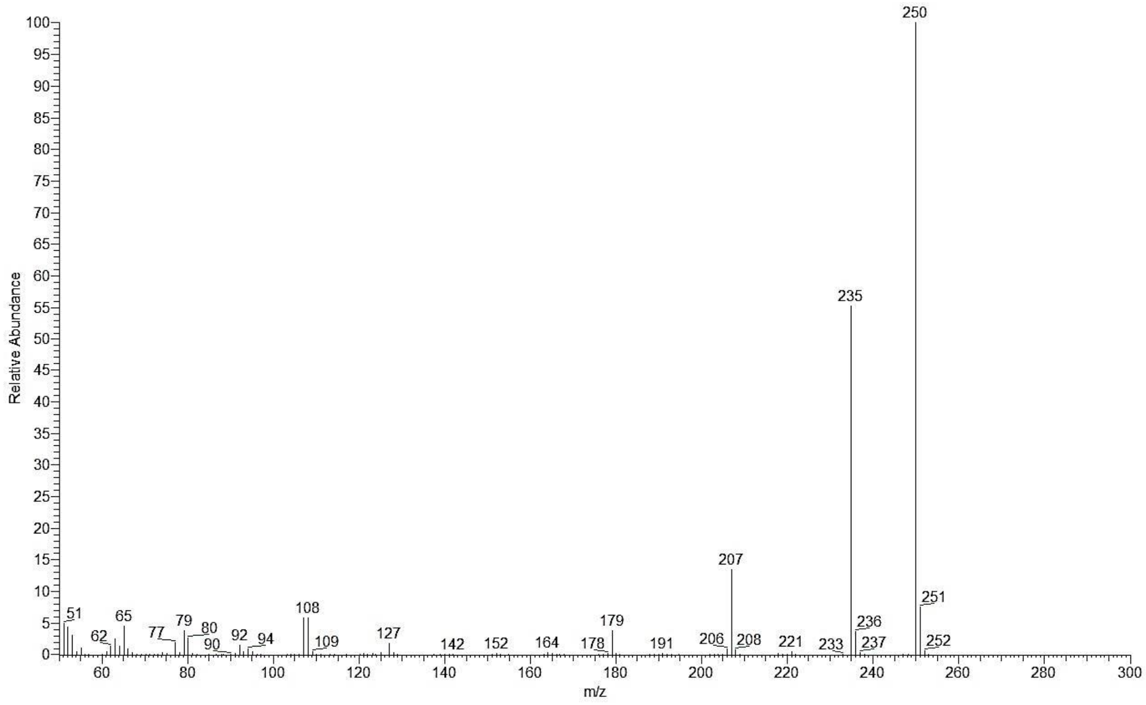

5-Iodoguaiacol (entry 13)

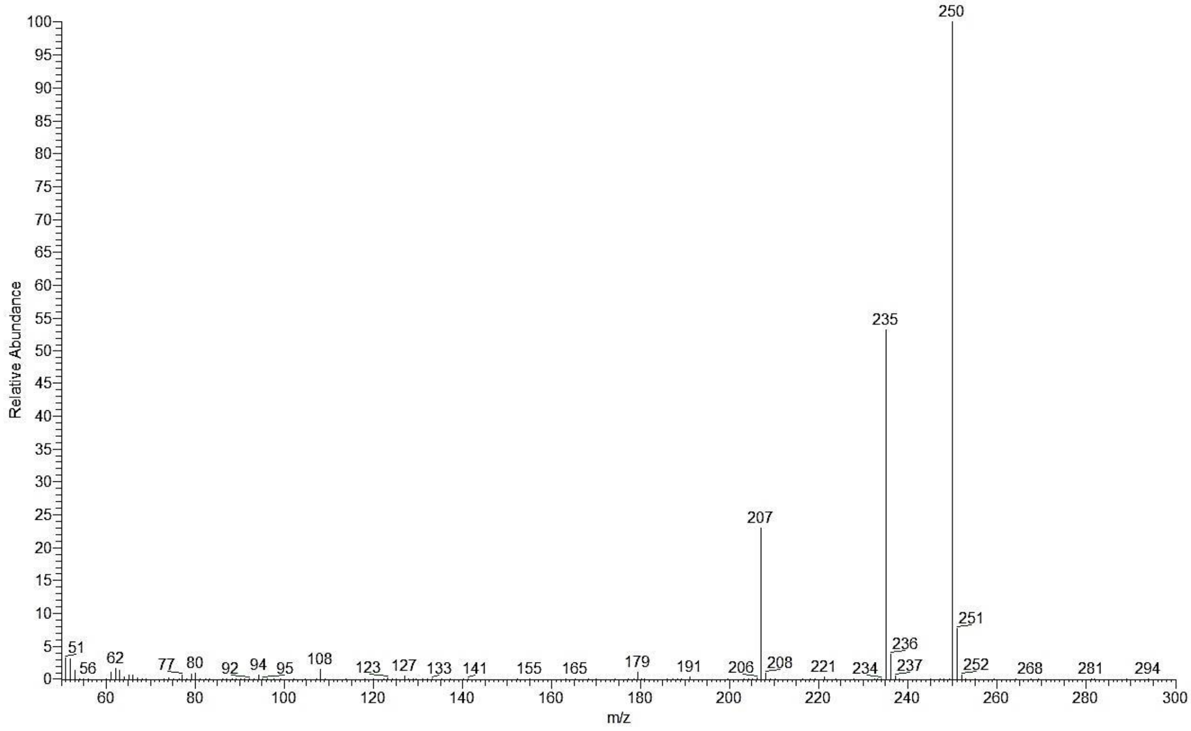

6-Iodoguaiacol (entry 14)

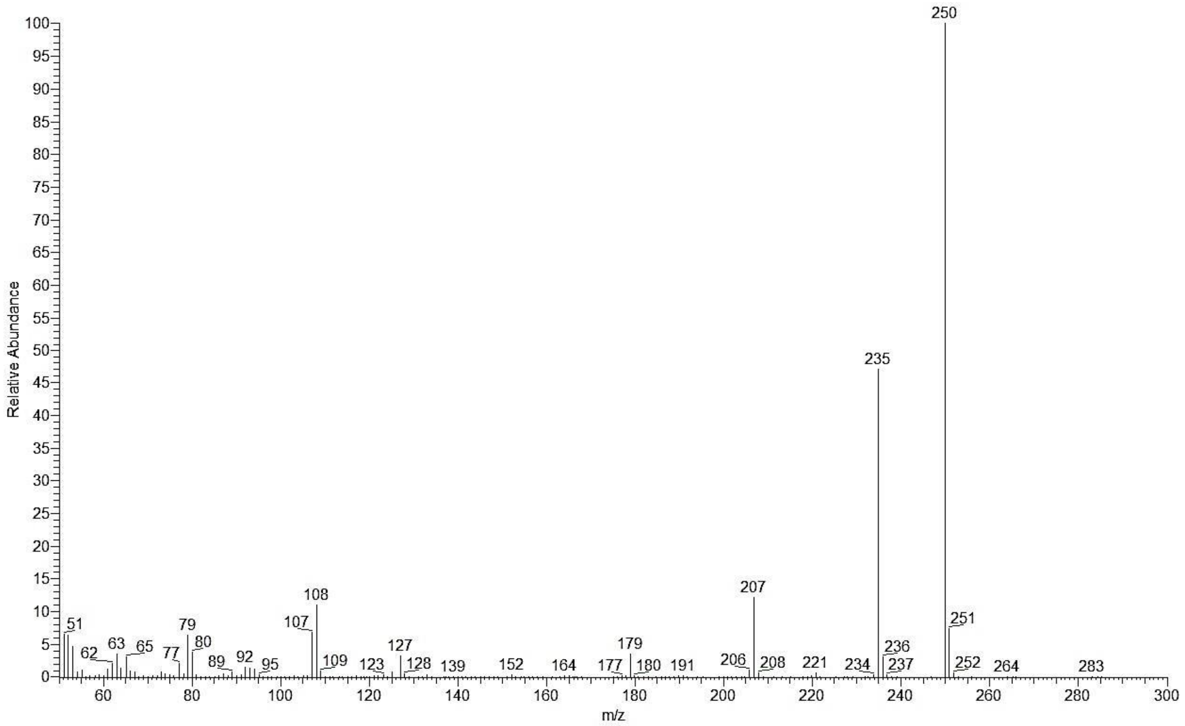

Table S1. Removal and degradation approaches of chlorinated guaiacol derivatives reported in literature

**Compound/s**	**Removed or degraded by**
4-Chloroguaiacol (19.7 %)<br>4,5-dichloroguaiacol (13.5 %)	Poly-aluminum chloride as coagulant[a]
4-Chloroguaiacol	Partly oxidized by UV irradiation and hydroxyl radicals
4-Chloroguaiacol	High surface area palm oil shell-activated carbon activated with NaOH[c]
4,5-Dichloroguaiacol	Peroxidase conjugate of cellulose nanocrystals
4,5-Dichloroguaiacol	Soil microorganisms
4-Chloroguaiacol<br>5-Chloroguaiacol<br>6-Chloroguaiacol<br>4,5-Dichloroguaiacol	Metabolized by *Acinetobacter junii* strain[f]
5-Chloroguaiacol (100 %)<br>4,5-Dichloroguaiacol (55 %)	Fungal treatment of extraction-stage effluent from chlorine bleaching kraft pulp with *Rhizopus oryzae*[g]
4-Chloroguaiacol (completely)<br>5-Chloroguaiacol (completely)<br>6-Chloroguaiacol (completely)<br>4,5-Dichloroguaiacol (partly)	Metabolization by *Rhodococcus ruber CA16* (soil bacterium)[h]

[a] (Choudhary et al., 2015)
[b] (Benitez et al., 2001)
[c] (Hamad et al., 2010)
[d] (Yang et al., 2008)
[e] (González et al., 1995)
[f] (González et al., 1993)
[g] (Nagarathnamma and Bajpai, 1999)
[h] (Acevedo et al., 1995)

Table S2. Odor qualities of all investigated halogenated guaiacol derivatives as reported by all 5 panelists

Entry	Odorant	P 1	P 2	P 3	P 4	P 5
1	3-Chloroguaiacol	smoky	musty, smoky	medical	smoky, medical	plastic-, vanilla-like
2	4-Chloroguaiacol	sweet, vanilla-like	whiskey-like, sweet	vanilla-like, sweet, smoky	sweet, vanilla-, marshmallow-like	sweet, vanilla-like
3	5-Chloroguaiacol	ham-like, smoked	sweet, smoky	smoky, clove-like	smoked, medical	smoked, ham-like
4	6-Chloroguaiacol	smoky, sweet	smoky, sweet, vanilla-like	vanilla-like, smoky, sweet, clove-like	smoky, ham-, pool-, plaster-like, medicinal	artificial, plastic-, ham-like, pungent
5	4,5-Dichloroguaiacol	smoky, sweet	sweet, smoky	smoky, clove-, vanilla-like	sweet, vanilla-like	clove-, vanilla-like
6	5,6-Dichloroguaiacol	smoky	vanilla-, plaster-like	smoky	medical, plaster-like	medical, plastic-like
7	3-Bromoguaiacol	medical	musty, old	musty	old	plastic-like, sweet
8	4-Bromoguaiacol	smoky, sweet	sweet, smoky, fruity	sweet, vanilla-like, musty	vanilla-like	vanilla-like
9	5-Bromoguaiacol	smoked	sweet, smoky, plaster-like	smoky, clove-like	sweet, smoky, medical	smoky, vanilla-like, sweet
10	6-Bromoguaiacol	musty, smoky	smoky, plaster-like	plaster-, plastic-like, burnt	medical, plaster-like	medical, plastic-like
11	3-Iodoguaiacol	smoky, burnt	musty, moldy	musty	musty	musty, moldy
12	4-Iodoguaiacol	smoky	smoky, vanilla-like	vanilla-, clove-like, smoky	sweet, vanilla-like	vanilla-like, sweet
13	5-Iodoguaiacol	smoked, sweet	sweet, vanilla-like	clove-like, sweet	smoked, sweet	sweet, chewing gum-like, smoked
14	6-Iodoguaiacol	smoked	smoky	n.p.	medical, plaster-like	medical, plastic-like

n.p.: Panelist was unable to perceive the odorant.

Table S3: Entry numbers, trivial and IUPAC names, structures and concentrations of FD1 solutions and IST (internal standard) in µg/mL.

Entry	Trivial name	IUPAC name	Structure	FD1 Conc. [µg/mL]	IST Conc. [µg/mL]
**1**	3-Chloroguaiacol	3-chloro-2-methoxyphenol	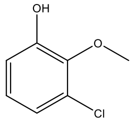	11.80	11.17
**2**	4-Chloroguaiacol	4-chloro-2-methoxyphenol	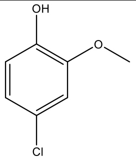	11.51	11.17
**3**	5-Chloroguaiacol	5-chloro-2-methoxyphenol	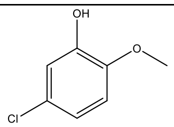	12.22	11.17
**4**	6-Chloroguaiacol	2-chloro-6-methoxyphenol	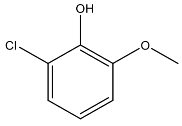	4.96	10.44
**5**	4,5-Dichloroguaiacol	4,5-dichloro-2-methoxyphenol	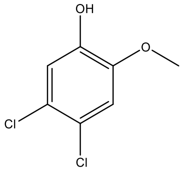	10.56	11.17
**6**	5,6-Dichloroguaiacol	2,3-dichloro-6-methoxyphenol	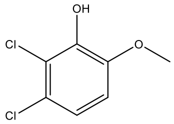	14.48	11.17
**7**	3-Bromoguaiacol	3-bromo-2-methoxyphenol	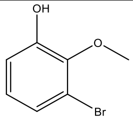	11.04	11.17
**8**	4-Bromoguaiacol	4-bromo-2-methoxyphenol	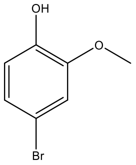	15.52	11.17
**9**	5-Bromoguaiacol	5-bromo-2-methoxyphenol	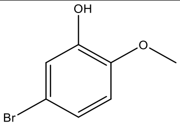	9.64	11.17
**10**	6-Bromoguaiacol	2-bromo-6-methoxyphenol	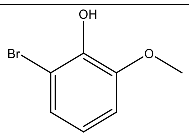	9.77	11.17

Table S3: Entry numbers, trivial and IUPAC names, structures and concentrations of FD1 solutions and IST (internal standard) in µg/mL, continued.

Entry	Trivial name	IUPAC name	Structure	FD1 Conc. [µg/mL]	IST Conc. [µg/mL]
**11**	3-Iodoguaiacol	3-iodo-2-methoxyphenol	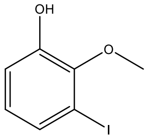	11.85	11.17
**12**	4-Iodoguaiacol	4-iodo-2-methoxyphenol	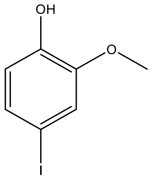	8.38	11.17
**13**	5-Iodoguaiacol	5-iodo-2-methoxyphenol	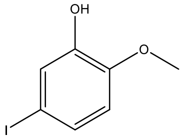	10.23	11.17
**14**	6-Iodoguaiacol	2-iodo-6-methoxyphenol	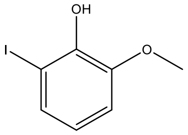	9.66	11.17

Figure S6: Determined odor thresholds for all investigated halogenated guaiacol derivatives, individual values of all panelists

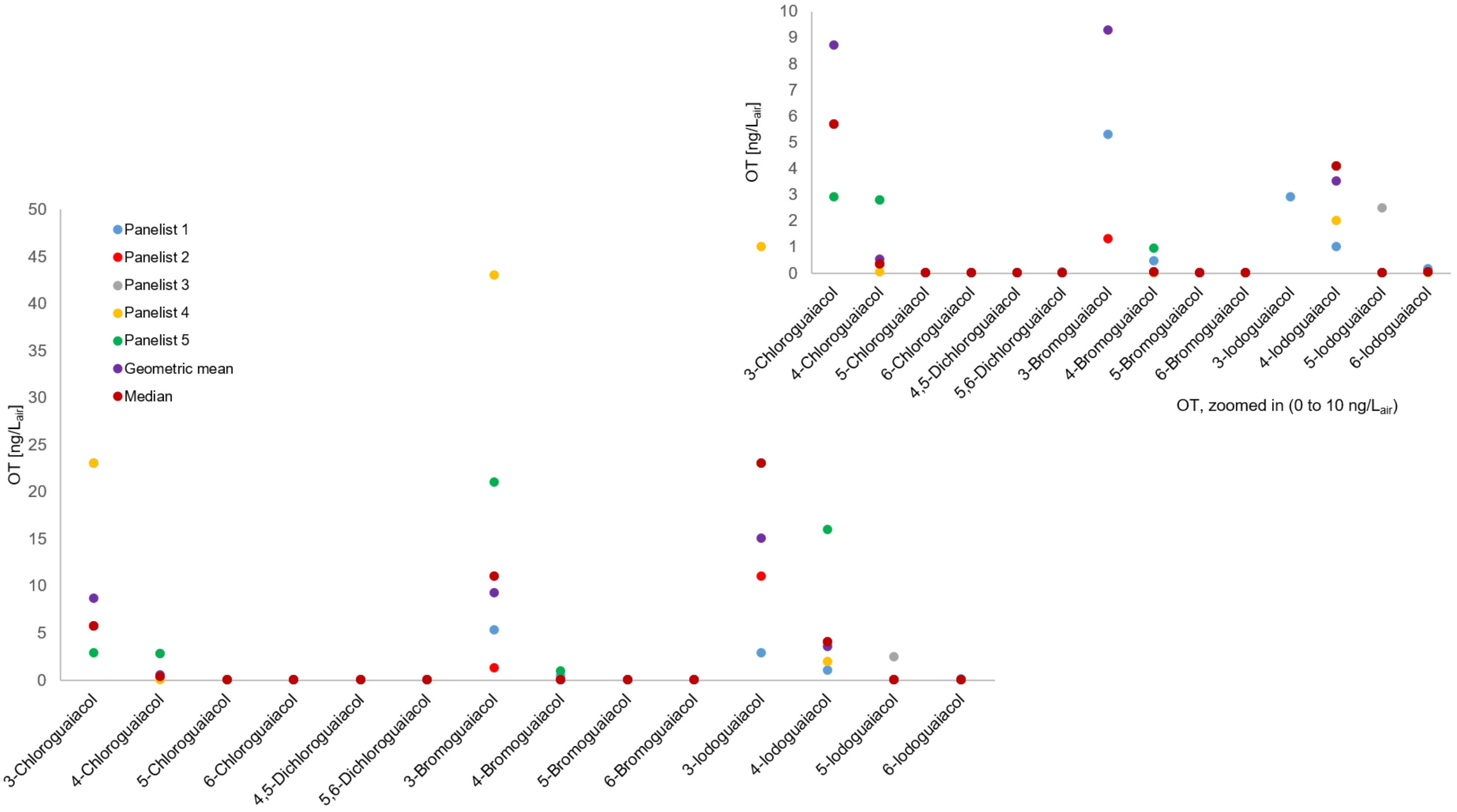

Figure S7: Determined odor thresholds for all investigated halogenated guaiacol derivatives, individual values of all panelists, logarithmic scale

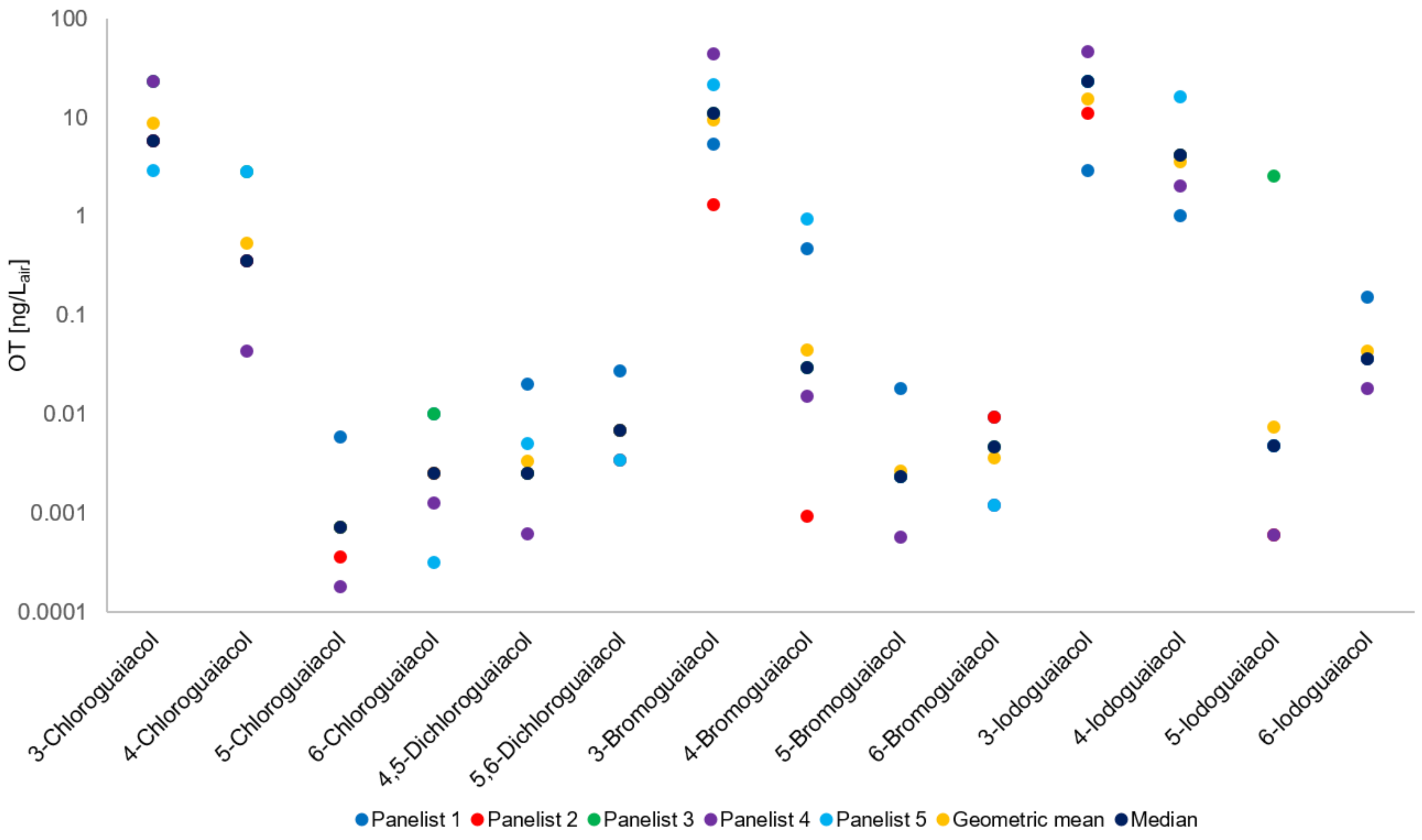

Figure S8: Boxplot of the odor thresholds for all investigated halogenated guaiacol derivatives (5 panelists, two males, three females), logarithmic scale. Mean value (± SD), markers at minimum and maximum OT, box perc. 25-75%.

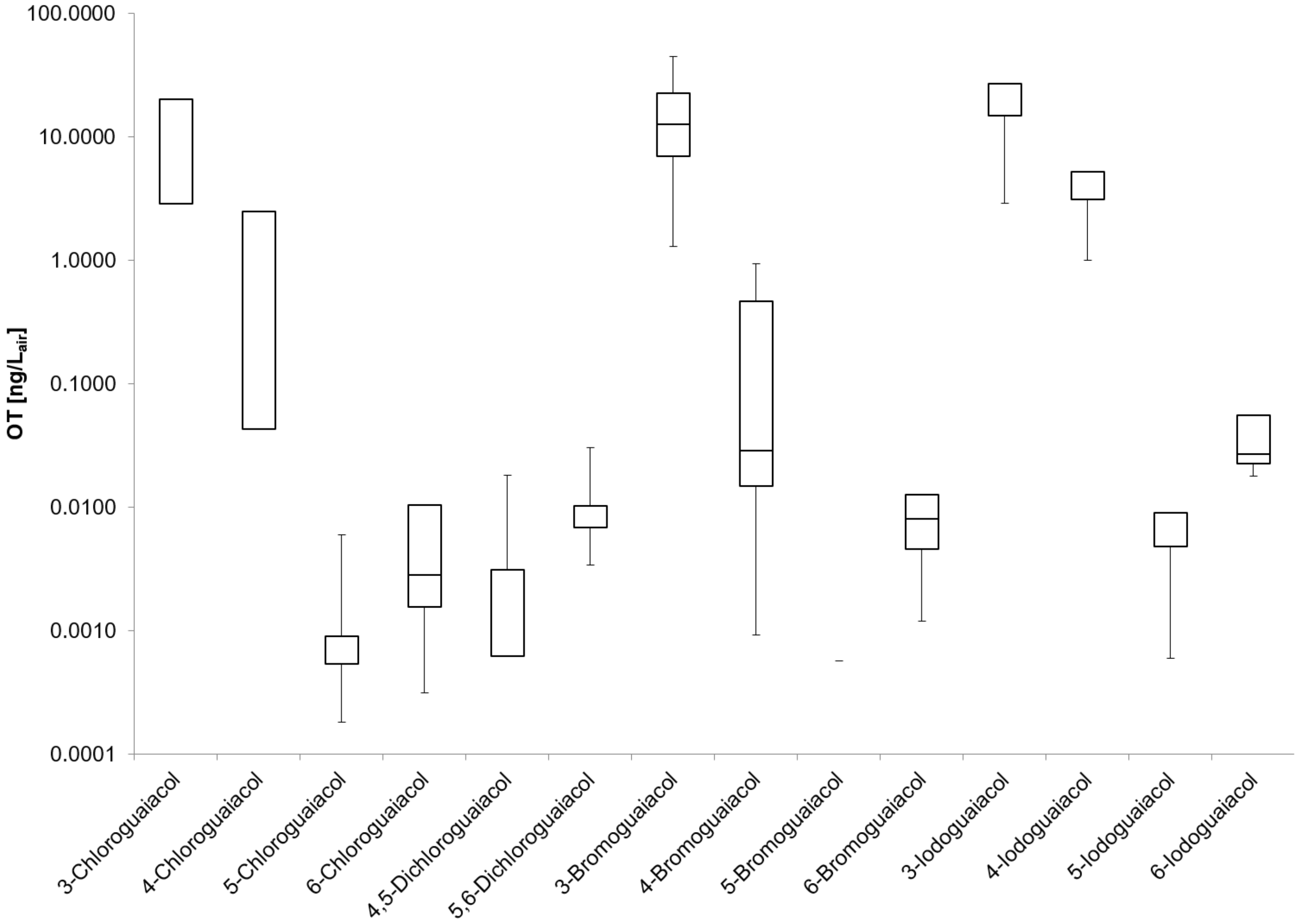

Acevedo, C., Brezny, R., Joyce, T.W., and González, B. (1995). Metabolism of mono- and dichlorinated guaiacols by Rhodococcus ruber CA16. *Current Microbiology* 30**,** 63-67.

Banwell, M.G., Hamel, E., Hockless, D.C.R., Verdier-Pinard, P., Willis, A.C., and Wong, D.J. (2006). 4,5-Diaryl-1H-pyrrole-2-carboxylates as combretastatin A-4/lamellarin T hybrids: Synthesis and evaluation as anti-mitotic and cytotoxic agents. *Bioorganic & Medicinal Chemistry* 14**,** 4627-4638.

Benitez, F.J., Beltran-Heredia, J., Acero, J.L., and Rubio, F.J. (2001). Oxidation of several chlorophenolic derivatives by UV irradiation and hydroxyl radicals. *Journal of Chemical Technology & Biotechnology* 76**,** 312-320.

Brady, R.M., Hatzis, E., Connor, T., Street, I.P., Baell, J.B., and Lessene, G. (2012). Synthesis of conformationally constrained benzoylureas as BH3-mimetics. *Organic & Biomolecular Chemistry* 10**,** 5230-5237.

Choudhary, A.K., Kumar, S., and Sharma, C. (2015). Removal of chloro-organics and color from pulp and paper mill wastewater by polyaluminium chloride as coagulant. *Desalination and Water Treatment* 53**,** 697-708.

Erickson, P.R., Grandbois, M., Arnold, W.A., and Mcneill, K. (2012). Photochemical Formation of Brominated Dioxins and Other Products of Concern from Hydroxylated Polybrominated Diphenyl Ethers (OH-PBDEs). *Environmental Science & Technology* 46**,** 8174-8180.

González, B., Acevedo, C., Brezny, R., and Joyce, T. (1993). Metabolism of chlorinated guaiacols by a guaiacol-degrading Acinetobacter junii strain. *Applied and Environmental Microbiology* 59**,** 3424-3429.

González, B., Brezny, R., Herrera, M., and Joyce, T.W. (1995). Degradation of 4,5-dichloroguaiacol by soil microorganisms. *World Journal of Microbiology and Biotechnology* 11**,** 536-540.

Hamad, B.K., Noor, A.M., Afida, A.R., and Mohd Asri, M.N. (2010). High removal of 4-chloroguaiacol by high surface area of oil palm shell-activated carbon activated with NaOH from aqueous solution. *Desalination* 257**,** 1-7.

Nagarathnamma, R., and Bajpai, P. (1999). Decolorization and Detoxification of Extraction-Stage Effluent from Chlorine Bleaching of Kraft Pulp by Rhizopus oryzae. *Applied and Environmental Microbiology* 65**,** 1078-1082.

Steen, P.O., Grandbois, M., Mcneill, K., and Arnold, W.A. (2009). Photochemical Formation of Halogenated Dioxins from Hydroxylated Polybrominated Diphenyl Ethers (OH-PBDEs) and Chlorinated Derivatives (OH-PBCDEs). *Environmental Science & Technology* 43**,** 4405-4411.

Weinstabl, H., Suhartono, M., Qureshi, Z., and Lautens, M. (2013). Total Synthesis of (+)-Linoxepin by Utilizing the Catellani Reaction. *Angewandte Chemie International Edition* 52**,** 5305-5308.

Yang, R., Tan, H., Wei, F., and Wang, S. (2008). Peroxidase conjugate of cellulose nanocrystals for the removal of chlorinated phenolic compounds in aqueous solution. *Biotechnology (Faisalabad, Pak.)* 7**,** 233-241.
